# Broad-spectrum metastasis suppressing compounds and therapeutic uses thereof in human tumors

**DOI:** 10.1038/s41598-023-47478-x

**Published:** 2023-11-21

**Authors:** Pnina Gottfried Komlosh, Jonathan L. Chen, Jessica Childs-Disney, Matthew D. Disney, Dan Canaani

**Affiliations:** 1 Department of Biochemistry and Molecular Biology, George Wise Faculty of Life Sciences, Tel Aviv University, 69978 Ramat Aviv, Israel; 2 Department of Biochemistry and Biophysics, University of Rochester Medical Center, 601 Elmwood Ave., Box 712, Rochester, NY 14642 USA; 3 Department of Chemistry, The Scripps Research Institute & UF Scripps Biomedical Research, 130 Scripps Way, Jupiter, FL 33458 USA

**Keywords:** Cancer, Molecular biology

## Abstract

Previously, we have identified a novel human metastasis-inducing lncRNA (named SKAI1BC), that suppresses the KAI1/CD82 metastasis-suppressing gene and is upregulated in triple negative breast cancer and melanoma derived cell lines. Modeling of the SKAI1BC lncRNA secondary structure and its potential interaction with Inforna compounds, led us to identify several compounds that might bind the SKAI1BC lncRNA. We found that these compounds inhibit metastasis invasion and cell migration in culture, in all eight types of solid human cancers tested: several of which are the most lethal and/or frequent human malignancies. Moreover, in most cases, the mechanism of action of several of our compounds involves enhancement of KAI1/CD82 RNA level depending on the specific compound and the human tumor type. With the epigenetic inactivation of KAI1/CD82 in at least ten additional solid human cancers, this implies a very good chance to broaden the spectrum of human cancers affected by our compounds. This is the first time that modeling of a large lncRNA (> 700 bp) secondary structure followed by its potential interaction with Inforna like compounds database has led to the identification of potential biologically active small molecule drugs.

## Introduction

One of the most unexpected findings of the genomics area is the extensive transcription of RNA from non-protein coding regions of the genome. Large-scale sequencing of human cDNA libraries elucidated tens of thousands of noncoding RNAs (ncRNAs), prominent among which are long noncoding RNAs (lncRNAs), or noncoding RNAs longer than 200 bp. The FANTOM CAT database of human lncRNAs with accurate 5′ ends currently contains 27,919 lncRNAs, out of which 19,175 are potentially functional. In turn, the LNCIPEDIA database currently has 127,802 human lncRNAs encoded by 56,946 genes. At least 1555 human lncRNAs have experimental evidence to be functional^1^. Thus, the function of the vast majority of the lncRNAs has not been investigated yet.

Several authors have shown initially the potential of promoter-directed short hairpin RNAs (shRNAs) to activate gene expression of tumor/metastasis suppressor genes silenced by promoter spanning as-lncRNAs^2,3^.

Two other prominent examples of breast cancer linked lncRNAs are HOTAIR (HOX antisense intergenic RNA) and MALAT1 (Metastasis-associated lung adenocarcinoma transcript 1). HOTAIR is a lncRNA that acts as a scaffold molecule by interacting with a chromatin modification complex that enables the HOXD gene silencing in trans. In primary and metastatic breast cancer cells, HOTAIR expression is up to hundreds-fold higher than in normal breast epithelia. This leads to transcriptional silencing of metastasis suppressor genes and results in tumor metastasis^4,5^. MALAT1, an abundant nuclear lncRNA, promotes cancer cell proliferation and metastasis in non-small cell lung carcinoma^4^.

A direct correlation of overall patients’ survival and KAI1 expression had been observed in at least the following five solid tumors: colorectal carcinoma, gastric carcinoma, non-small cell lung cancer (NSCLC), breast cancer, and laryngeal squamous cell carcinoma (LSCC), reviewed in^6^. Likewise, at least 10 other human solid tumors are deficient in KAI1/CD82 metastasis suppressor gene expression: hepatocellular carcinoma, clear cell renal cell carcinoma, melanoma, osteosarcoma, pancreatic carcinoma, prostate cancer, ovarian cancer, bladder carcinoma, cervical carcinoma, and thyroid cancer^6–9^.

Noteworthy, in at least three solid tumors (gastric, cervical, and ovarian cancers) KAI1 affects also tumor proliferation^7^. At the transcriptional level KAI1 is upregulated by several transcription factors such as AP2, p53, JunB, and ΔNp63α^7^. Post transcriptionally, in liver HCC cells and gastric stomach cancer KAI1 is negatively regulated by miR-197 and miR-362-3p. Similarly, miR-338-5p negatively regulate KAI1 RNA in melanoma A375 cells while miR-217 suppresses KAI1 expression in NSCLC. Loss of heterozygosity (LOH) of KAI1 in human cancers is a rare event, and similarly no point mutations were found in the KAI1 gene in human malignancies^7^. Thus, KAI1 expression in human tumors is clearly being epigenetically silenced. The promotion of homotypic cell–cell adhesion is an important metastasis suppressive function of KAI1.

Recent reports propose a combination of altered KAI1-protein interactions and signaling pathways. It has been suggested that KAI1 interacts directly with the epidermal growth factor receptor (EGFR) and weakens migration signaling by rapid desensitization of EGF-induced signals^7^. In addition, the actin cytoskeleton organizing FAK-Lynp130^CAS^-CrkII pathway is attenuated by KAI1 mediated inhibition of the active p130^CAS^-CrkII complex formation.

In order to try identifying new breast cancer affecting lncRNA(s), we established a primer-specific RT-PCR screening process for promoter-spanning lncRNAs of antisense orientation^10^. For that purpose, total cellular RNA/nuclear RNA from three different TNBC cell lines, (MDA-MB 231, Hs578T and SUM149PT) were subject to RT-PCR with primers specific for potential upstream promoter-spanning antisense RNAs for ten of the most important human breast metastasis suppressors- and tumor suppressor- genes (Maspin, CST6, RAR-β, SYK, MAL, VGF, OGDHL, KIF1A, FKBP4, and KAI1/CD82). Noteworthy, out of these 10 tested genes, only KAI1/CD82 had an antisense lncRNA spanning its promoter and suppressing it in the triple-negative breast cancer cell line MDA-MB-231 cell line. The existence of a KAI1 antisense lncRNA that is transcribed off the KAI1/CD82 promoter imply the latter is a bidirectional promoter, a finding that we previously reported^11^. We found that the KAI1 as-lncRNA is not polyadenylated, and is primarily present in the nucleus. Moreover, specific degradation of this lncRNA via RNAi (with either constitutive or inducible shRNAs expression) in breast cancer cells or melanoma cells led to a drop in this lncRNA and increase in KAI1 RNA and protein level in both breast cancer MDA-MB-231 and MDA-MB-435 melanoma cells. This leads to increase in cell adherence and consequently, inhibition of cancer cell invasiveness and cell migration^11^. Therefore, this lncRNA is a natural suppressor of KAI1 and consequently an enhancer of the metastasis process. Accordingly, we named it SKAI1BC-"Suppressor of KAI1 in Breast Cancer". Sequencing of this lncRNA showed that it is 792 bp long^11^. An identical sequence of a human long noncoding RNA of 792 bases (without a 3’ polyA tail) derived from a cDNA library of the GM12878 cell line, contig_343318, was then found in the UCSC database as UCSC Accession no. wgEncodeEH000148, one of the many thousands of transcripts derived off this cell line. However, no other information was reported about this RNA transcript.

Previously we also disrupted SKAI1BC RNA activity by oligonucleotides (modified and non-modified) in the form of ASO or siRNA^11^. However, despite numerous attempts over the past 43 years (oligonucleotides), and 24 years (RNAi based technologies), the FDA approved only 4 ASOs and 11 siRNAs as drugs^12^. Therefore, we decided to try to ablate the oncogenic activity of SKAI1BC lncRNA, via RNA-binding small molecules^13^. Previously we developed a method to predict small compounds binding to RNAs^14,15^. We have used this approach to identify small molecules that target expanded repeating RNAs, which cause or contribute to neurological/neuromuscular diseases, as well as small compounds that target oncogenic pre-miRNAs and viral RNA^14,16–18^. Until 1/1/2021, the FDA has so far approved one compound (ridisplam, PTC/Roche) as a drug^19^. Therefore, there is an urgent need to provide effective compounds for inhibiting metastatic properties of cancer cells^13,20^.

## Results

One acute problem in cancer therapy is the scarcity of effective drugs against metastasis, resulting in 90% of cancer deaths. Another major difficulty emerging in cancer therapy is the recent realization that many of the ~ 30,000 unexplored human lncRNAs are functional and a good many are likely to be involved in malignancy. Our recent discovery of a novel human lncRNA, which we initially identified as a suppressor of the KAI1/CD82 in triple-negative breast cancer^11^, confronted us with the question of how to stimulate the epigenetically suppressed activity of KAI1 in metastatic cancer(s). This is a challenging problem because the KAI1/CD82 metastasis suppressor is known to be epigenetically silenced in at least 15 solid human tumors and possibly a few hematopoietic human malignancies. Importantly, the level of KAI1 expression is prognostic of overall survival or other clinical key features of the patients’ disease in at least 10 solid human cancers.

### Predicting the structure of the SKAI1BC lncRNA and computational screening for potential KAI1 as-lncRNA binding molecules

To assess the drugability of the 792 bp long KAI1 as-lncRNA, we used ScanFold 2.0 to identify regions of the RNA likely to form functional structures. First, the RNA was folded with ScanFold-Scan in 120 nt windows to find local structural motifs. The 120 nt window size was chosen on the basis that previous studies found a window size between 100 and 150 nt to be optimal^21^. For each window, ScanFold-Scan calculates a thermodynamic z-score, an indication of unusual structural stability of an RNA, and the ensemble diversity (ED), a measure of diversity of structures in an ensemble. The average z-score for all windows is − 0.67 ± 1.16, indicating that the RNA can potentially fold into stable structures. Among the 673 windows, 119 have z-scores more than one standard deviation below the average z-score for all windows. The average ED for all windows is 25.62 ± 12.35, and 77 of the 119 lowest z-score windows have ED values more than one SD below average. ScanFold-Fold was then used to identify base pairs that generate low z-score windows.

**Figure 1:**
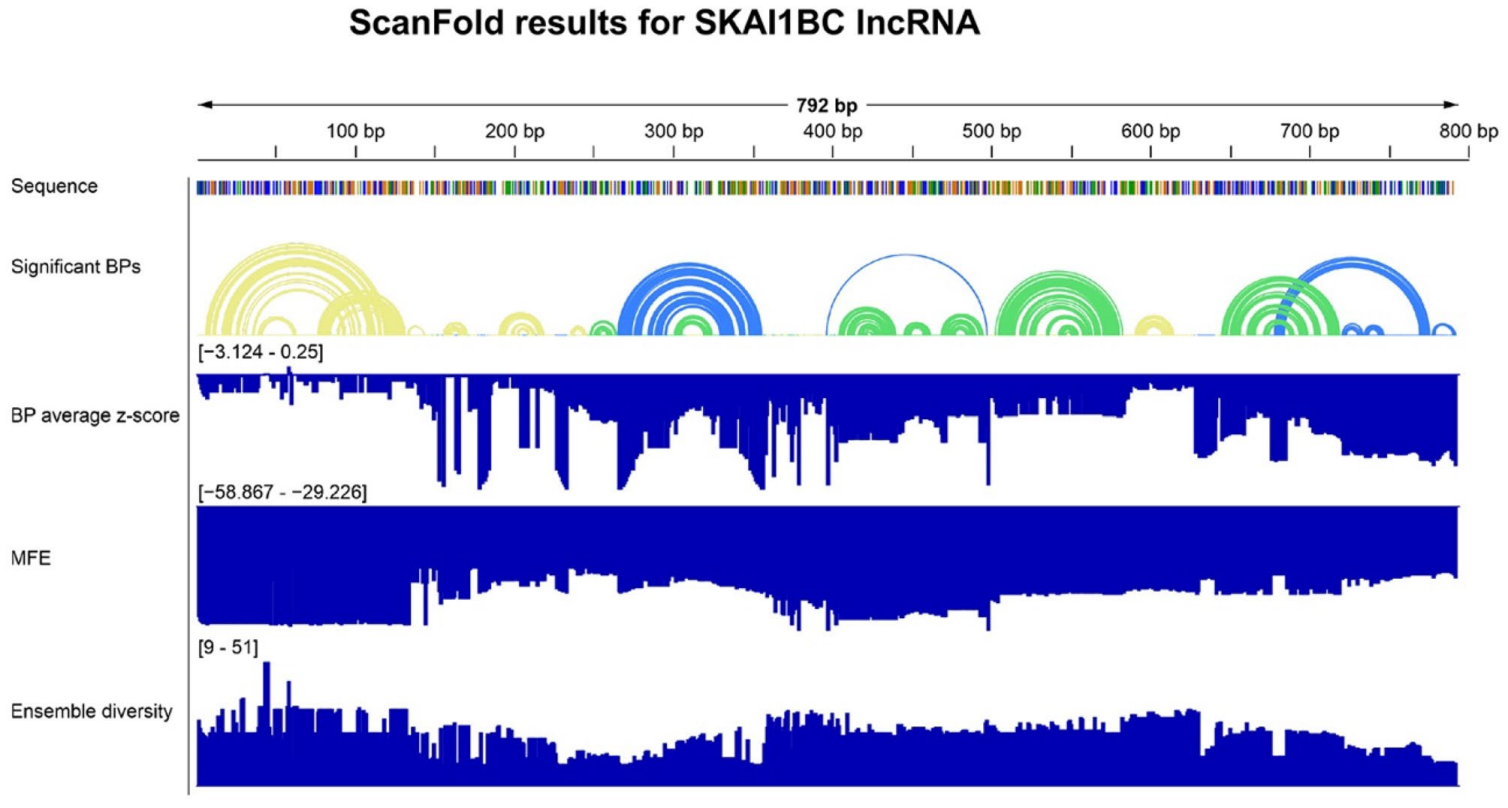
ScanFold results for SKAI1BC as-lncRNA. The base pair arc diagram, ΔG 37° z-score, MFE, and ensemble diversity values are shown. Arcs representing base pairs are colored according to their Z_avg_ score: yellow, Z_avg_ < 0; green, Z_avg_ <  − 1; blue, Z_avg_ <  − 2.

**Figure 2:**
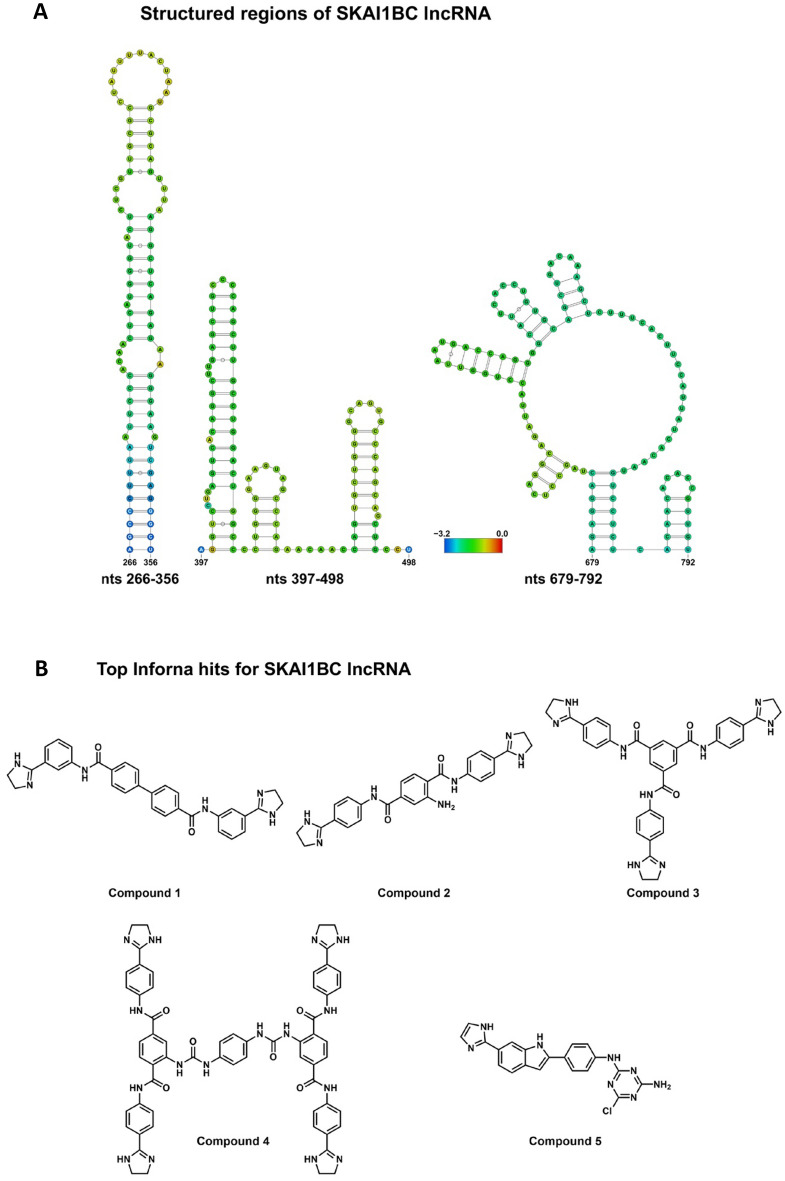
Structures of SKAI1BC as-lncRNA and Compounds tested against the SKAI1BC as-lncRNA. (**A**) Bases in the structures are colored by their Z_avg_, where blue indicates the most negative and red indicates the most positive. (**B**) Compounds 1–4 were predicted to bind to structures in the SKAI1BC as-lncRNA with high fitness scores and obtained from the NCI for use in this study. Compound 5 was not predicted to bind to any structural features in the as-lncRNA.

ScanFold-Fold predicted 192 base pairs, including 18 in two pseudoknots (Fig. 1). The number of base pairs was reduced to 133, including seven in a pseudoknot, when filtered for those with an average nucleotide z-score (Z_avg_) below − 1. Lowering the Z_avg_ cutoff to − 2 further decreased the number of base pairs to 42. The folding landscape is dominated by structures with Z_avg_ >  − 2, with structures with Z_avg_ <  − 2 dispersed throughout the lncRNA. Z_avg_ and ED correlate, as determined by a correlation coefficient of 0.75; base pairs with lower Z_avg_ values also have lower ED values. However, there does not appear to be a positive trend between either of these metrics and ΔG_37_°. Constrained refolding of structures containing Z_avg_ <  − 2 base pairs using RNAfold added 52 base pairs to the structures (Fig. 2A). ScanFold-Fold extracted motifs from these structures have z-scores and ED values ranging from − 5.20 to − 3.15 and 0.93 to 9.12, respectively (Table 1). In summary, stable structures were predicted throughout the SKAI1BC as-lncRNA.

**Table 1:** ΔG_37_°, Z-score and ED values of ScanFold-Fold extracted motifs.

Region (nts)	ΔG_37_° (kcal/mol)	Z-score	ED
267–356	− 36.2	− 4.80	4.64
398–498	− 48.7	− 3.15	5.63
680–776	− 30.1	− 3.18	9.12
779–792	− 1.60	− 5.20	0.93

These presumably targetable small structures were compared to the RNA motifs-small molecule interactions Inforna 2.0 database, which was generated experimentally primarily by one of us, M. Disney^15^. The Inforna search yielded four compounds (#1–#4) with fitness scores of at least 70% for each region, the top hits among which are shown in Fig. 2B. The 5′CAU/3′G_A and 5′CAC/3′G_G bulge loops in these regions were predicted to bind to a similar set of compounds with fitness scores up to 81.2%, indicating that a particular compound may be able to inhibit multiple sites on the human SKAI1BC lncRNA (Table 2). The compounds were tested together with another compound #5 that was not predicted to bind KAI1 as-lncRNA.

**Table 2:** Predicted binding sites of compounds in the SKAI1BC as-lncRNA.

Region (nts)	Motif	Motif probability (%)	Compound	Fitness score (%)
266–356	5′CAU/3′G_A	97.8	1	72.1
			2	83.0
			3	84.7
			4	81.2
397–498	5′CAC/3′G_G	99.4		71.0

Compound 5 was not predicted to bind to any structures in the SKAI1BC as-lncRNA.

### Testing the compounds on TNBC cell lines

We sought for compounds that not only bind the SKAI1BC as-lncRNA, but also inhibit its activity as might be reflected in KAI1 stimulation. Compounds #1–#5 are imidazole derivatives.

**Figure 3:**
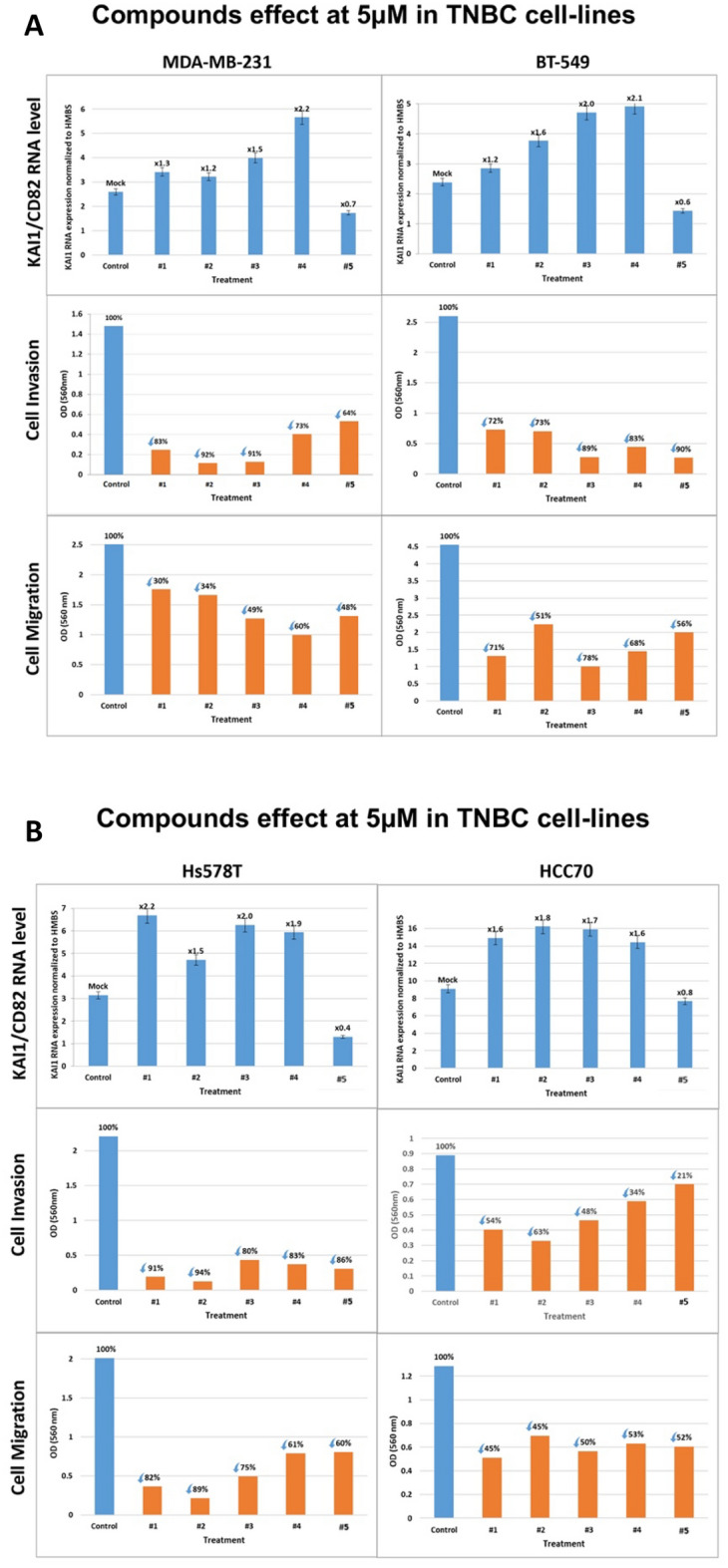

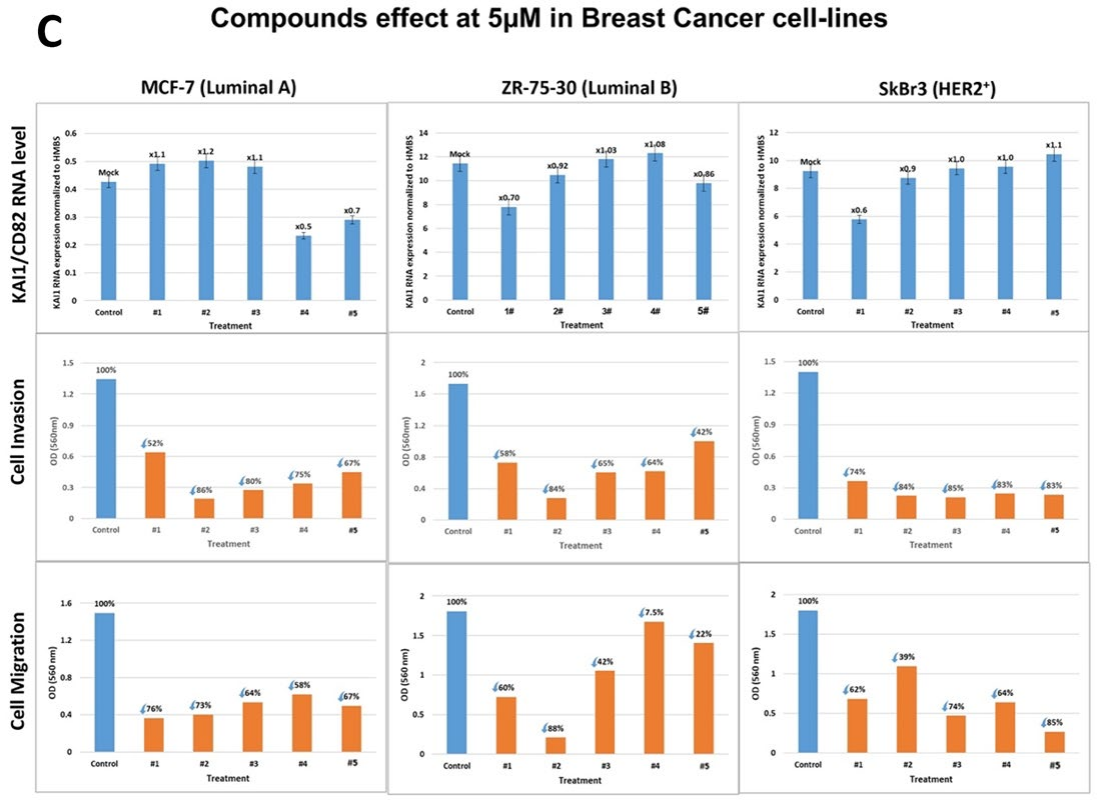
(**A**–**C**) Compounds effect on KAI1/CD82 RNA level, cell invasion and cell migration of TNBC and other breast cancer groups’ cell lines. (**A**) TNBC MDA-MB-231 and BT-549 cell lines. (**B**) TNBC Hs578T and HCC70 cell lines. (**C**) MCF-7 (Luminal A), ZR-75–30 (Luminal B), and SkBr3 (HER2 +) cell lines. Cells were treated with 5 µM of each of the compounds (#1–#5) for 48 h. Effect on KAI1 RNA level (upper rows**, A**, **B**, **C**): Cells were treated with the compounds for 48 h. Relative KAI1 mRNA expression was quantified by RT followed by Real-Time PCR with HMBS as endogenous control. Effect on Cell Invasion and Migration (middle and lower rows, **A**, **B**, and **C**, respectively): Cells were treated with the compounds for 24 h and then seeded for additional 24 h while under starvation conditions and in the presence of the compounds either in CytoSelect (Cell Biolabs) transwell chambers for cell invasion assay or in ThinCert™ (Greiner Bio-One) inserts for cell migration assay. Cells’ invasion or migration from the upper chamber to the chemoattractant (10% serum) in the lower chamber were quantified using colorimetric assay.

Therefore, compounds #1–#5 were first assayed at 5 µM concentration for stimulating the RNA level of the KAI1 metastasis suppressor in TNBC cell lines (Fig. 3A). The results show that compounds #1–#4 at 5 µM stimulated KAI1 RNA expression by up to 2.0–2.2-fold in the TNBC MDA-MB-231 and BT-549 (Fig. 3A ( upper row), HCC70, and Hs578T cell lines (Fig. 3B (upper row). The extent of KAI1 RNA stimulation was both compound- and cell line-dependent. The nonmetastatic MCF10A cell line served as the negative control to these experiments and did not respond at all (data not shown). According to the computational screening disclosed above, Compound #5, which belongs to a different chemical group and was predicted not to bind KAI1 as-lncRNA, did not stimulate KAI1 RNA level in the four TNBC cell lines (Fig. 3A,B). Moreover, incubation of these four compounds at 5 µM for 48 h with each of these TNBC cell lines resulted in severe inhibition of metastasis cell invasion, typically from 54 to 94% (Fig. 3A–B)**,** middle row). Interestingly, compound #5 inhibited cell invasion of all four TNBC cell lines, even though it did not boost KAI1 RNA level in MDA-MB-231, BT-549, HCC70 and Hs578T (Fig. 3A–B)**,** middle row). In contrast, none of the five compounds inhibited the very modest invasion of the MCF10A derived cells (data not shown). These experiments were followed by inquiry of the compounds effect on cell migration, the second step of metastasis following cell invasion. As shown (Fig. 3A–B, bottom row), cell migration in all four tested TNBC cell lines (MDA-MB-231, BT-549, HCC70 and Hs578T) was inhibited (from 30 to 89%) by each of the five compounds, including compound #5. Therefore, based on compound #5 reactions, inhibition of cell invasion and migration can be triggered not only by elevation of the KAI1 metastasis suppressor RNA, but also by other mechanism(s).

### Testing the compounds on non-TNBC breast cancer cell-lines

These initial results led us to test three non-TNBC breast cancer cell lines: MCF-7 (Luminal A), ZR-75-30 (Luminal B) and SkBr3 (HER2+) cell lines (Fig. 3C**,** upper row). In all three cell lines, there is no significant stimulation of KAI1 RNA level by each of the five compounds (#1–#5). The five compounds managed to inhibit cell invasion and cell migration in all three Breast cancer cell lines (Fig. 3C, middle and bottom rows). The lack of stimulation in KAI1 RNA level in compounds treated cells clearly demonstrates that depending on the cell line, inhibition of metastasis cell invasion and migration can occur even in the absence of the metastasis suppressor KAI1 RNA enhancement via a different mechanism.

### Testing the compounds on melanoma cell lines

**Figure 4:**
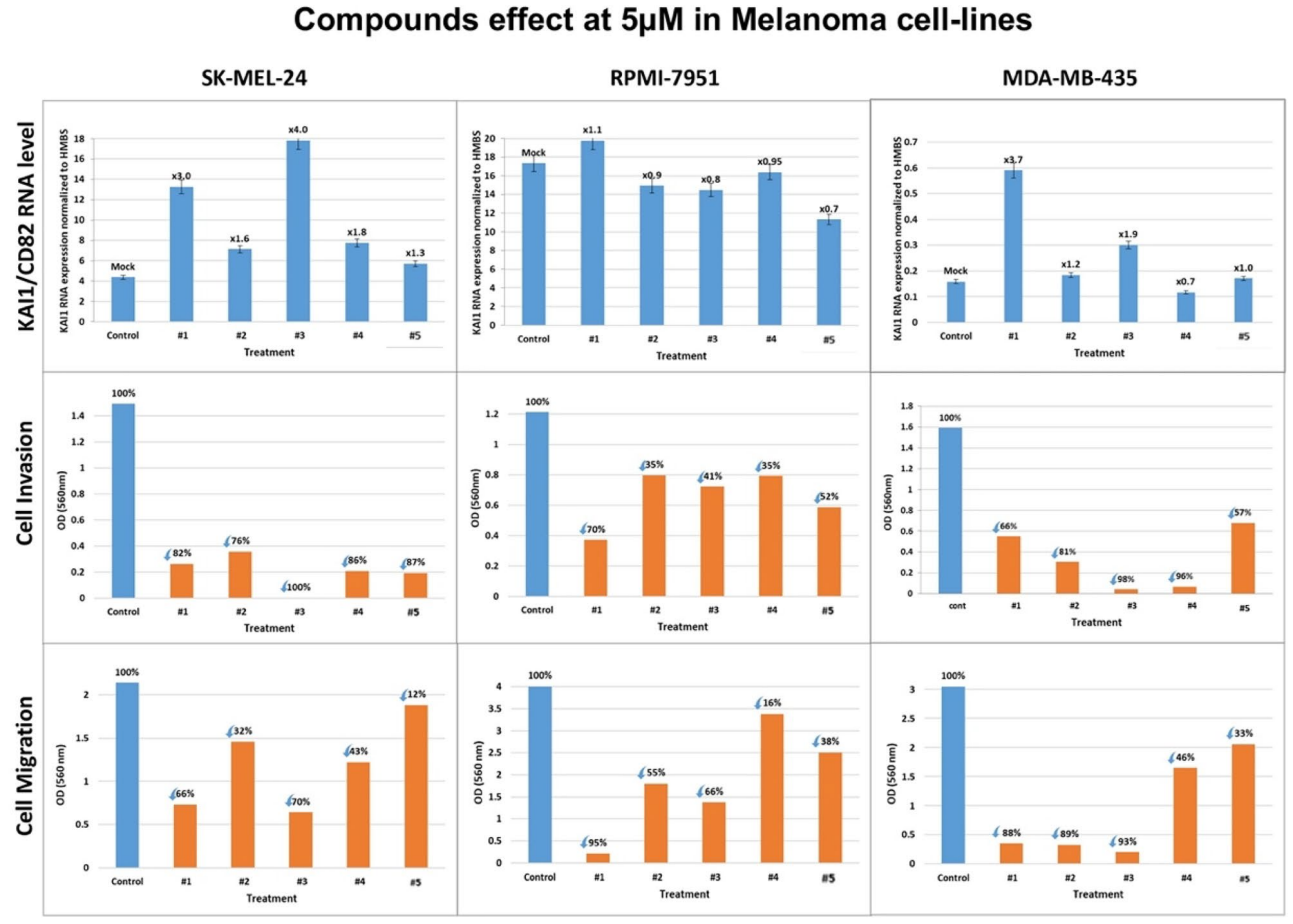
Compounds effect on KAI1CD82 RNA level, cell invasion and cell migration of Melanoma cell lines. SK-MEL-24, RPMI-7951 and MDA-MB-435 cells were treated with 5 µM of each of the compounds (#1-#5), for 48 h, and processed as outlined above. Effect on KAI1 RNA level (upper row). Effect on Cell Invasion and Migration (middle and lower rows**,** respectively).

In SK-MEL-24 and MDA-MB-435 cell lines (Fig. 4, upper row), there is significant stimulation of KAI1 RNA level by each of the four compounds (#1–#4). With compound #5 there is a minor elevation in KAI1 RNA level in SK-MEL-24. In the RPMI-7951 cell line, on the other hand, there is no KAI1 RNA stimulation (Fig. 4, upper row). The five compounds managed to inhibit cell invasion in all three melanoma cell lines including in RPMI-7951 (Fig. 4, middle row). Moreover, there was also cell migration inhibition triggered by all five compounds (Fig. 4, bottom row) in the melanoma cell lines.

The different pattern of KAI1 RNA level in compounds treated RPMI-7951 cells clearly demonstrates again that depending on the cell line, inhibition of metastasis cell invasion and migration can occur even in the absence of the KAI1/CD82 metastasis suppressor RNA enhancement, apparently via a different mechanism.

Interestingly, the concentration curve of molecule #2 in the melanoma MDA-MB-435 cells inhibited invasiveness by 81% at 5 µM, and by 23% at 50 nM; implying very potent cell invasion inhibition by this compound.

### Testing the compounds on NSCLC cell lines

**Figure 5:**
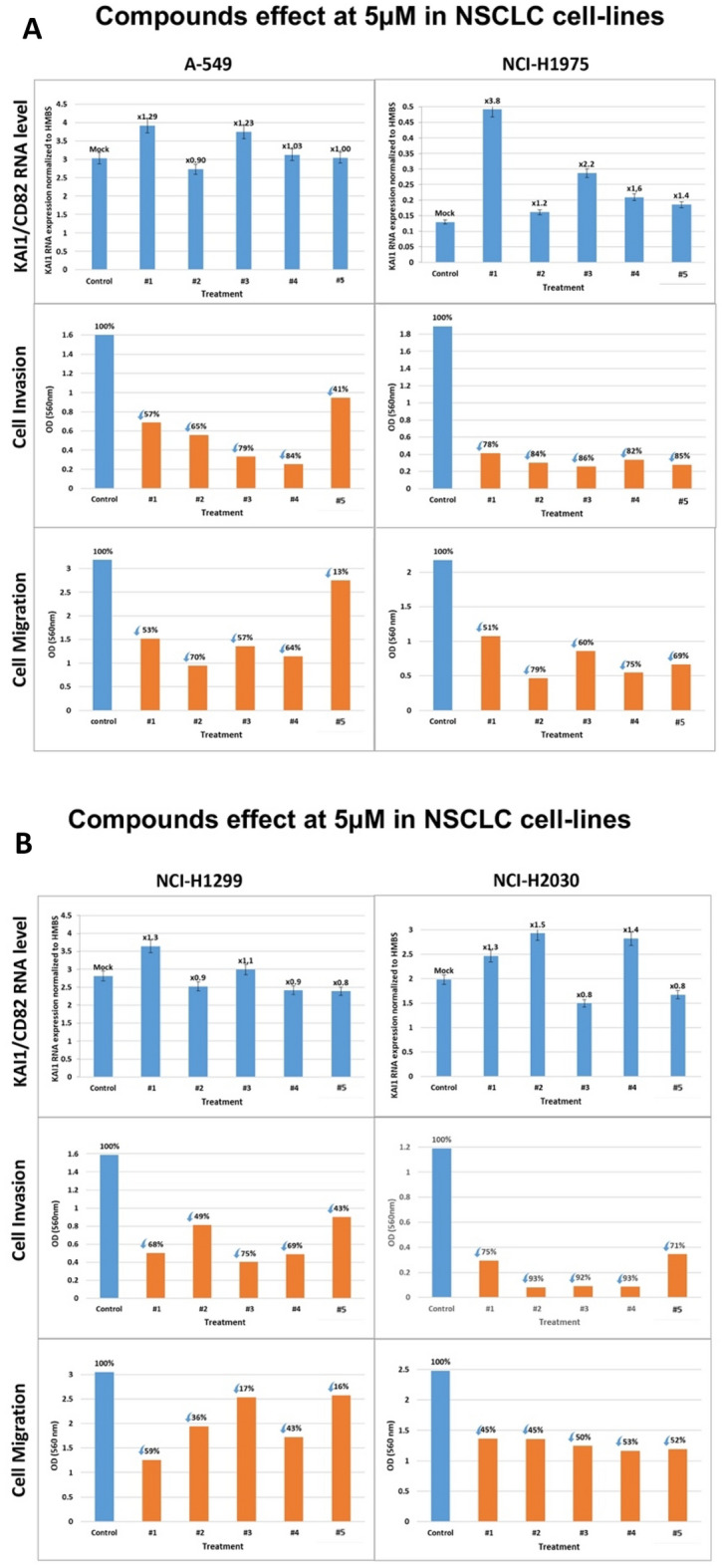
(**A**–**B**) Compounds effect on KAI1CD82 RNA level, cell invasion and cell migration of NSCLC cell lines. (**A**) A-549 and NCI-H1975 cell lines. (**B**) NCI-H1299 and NCI-H2030 cell lines. Cells were treated with 5 µM of each of the compounds (#1–#5), for 48 h, and processed as outlined above. Effect on KAI1 RNA level (upper rows, **A**, **B**). Effect on Cell Invasion and Migration (middle and lower rows, **A** and **B**, respectively).

A-549, NCI-H1975, NCI-H1299 and NCI-H2030 are the Non-Small Cell Lung Carcinoma (NSCLC) derived cell lines tested in this work. In general, the NCI-H1975 and NCIH2030 cell lines responded better to the compounds than the other two cell lines in terms of KAI1 RNA stimulation (Fig. 5A–B, upper row). Yet, all five compounds inhibited invasion and migration in all four-cell lines irrespective of whether they induced KAI1 RNA production (Fig. 5A–B, middle and bottom row). This observation again demonstrates that there are at least two anti-metastatic mechanisms shared by the five compounds: one, which acts via KAI1 RNA elevation, the other independent of KAI1 gene expression enhancement.

### Testing the compounds on pancreatic cancer cell lines

**Figure 6:**
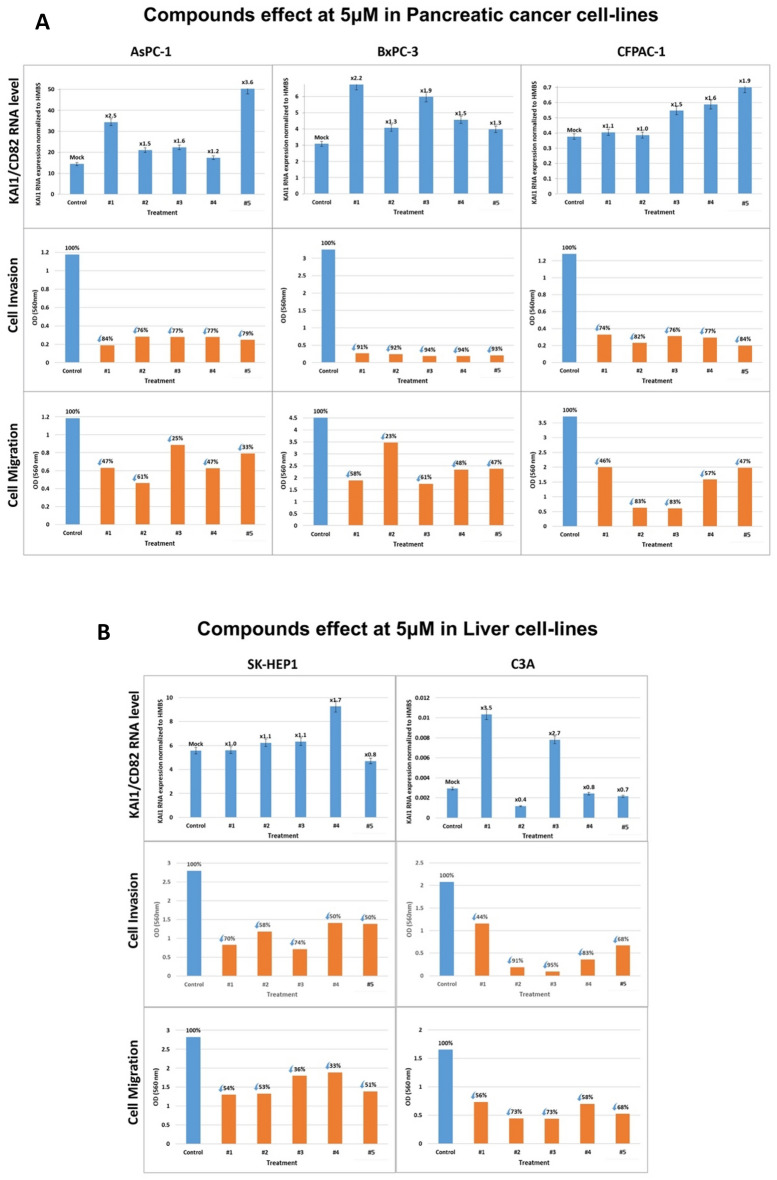
Compounds effect on KAI1CD82 RNA level, cell invasion and cell migration of pancreatic and liver cancer cell lines. (**A**) Pancreatic cancer cell lines: AsPC-1, BxPC-3 and CFPAC-1. (**B**) Liver carcinoma SK-HEP1 and C3A cell lines. Cells were treated with 5 µM of each of the compounds (#1–#5), for 48 h, and processed as outlined above. Effect on KAI1 RNA level (upper rows, **A**, **B**). Effect on Cell Invasion and Migration (middle and lower rows, **A** and **B**, respectively).

AsPC-1, BxPC-3 and CFPAC-1 are the three pancreatic carcinoma cell lines tested with the five compounds at 5 µM in this work (Fig. 6A). The KAI1 RNA level in AsPC-1 and BxPC-3 was elevated by all compounds, including compound #5, while only compounds #3–#5 stimulated the KAI1 RNA level in CFPAC-1 (Fig. 6A upper row). Yet, all compounds triggered significant metastasis cell invasion inhibition and cell migration inhibition in all three-cell lines (AsPC-1, BxPC-3, CFPAC-1, see Fig. 6A, middle and bottom rows). These results imply that, depending on the treated human tumor cell line, compound #5 may affect metastasis in vitro via KAI1 RNA enhancement as well.

### Testing the compounds on liver carcinoma derived cell lines

SK-HEP1 and C3A are the Liver Cell Carcinoma cell lines tested in this work. In the SK-HEP1 cell line, only compound #4 induced KAI1 RNA stimulation (by × 1.7), whereas in C3A compounds #1 and #3 stimulated KAI1 RNA by × 3.5 and × 2.7, respectively (Fig. 6B, upper row). Yet, all five compounds inhibited invasion and migration in both cell lines irrespective of whether they induced KAI1 RNA production or not (Fig. 6B, middle and bottom row). This observation again demonstrates that the five compounds could act via KAI1 RNA elevation or independently of KAI1 gene expression enhancement.

### Comparing the gene expression pathways affected in TNBC cells between compound #3 versus compound #5

Comparison of the genes expression pathways in TNBC MDA-MB-231 cells treated by 0.05% DMSO mock vs by Compound #5 revealed 12 pathways that are inhibited significantly by Compound #5 with FC -1.5 till -2.14, while the pValues are less than 0.05. Among these pathways are several known to participate in human metastasis, such as Cell Adhesion and TGF-beta.

The gene expression pathways were next compared between TNBC MDA-MB-231 cells treated by Compound #3 versus Compound #5. When one considers only the pathways in which the pValue is less than 0.05, there are 40 pathways that Compound #5 inhibits more than Compound #3, with FC ranging from − 1.5 till − 3.04. Among these pathways, are several known to participate in human metastasis, such as Cell Adhesion, mTOR signaling, PI3K-Akt signaling, and WNT signaling.

These data indicate that while Compounds #1–#4 suppress metastasis of TNBC cell lines via enhancement of KAI1/CD82 RNA (Fig. 3A and B), compound #5 acts through other membranes-associated pathway/s, as outlined above.

## Discussion

As discussed herein, the present disclosure relates to small molecules that inhibit solid tumors’ metastasis. Five compounds have been tested and proven active in cultures of eight solid human tumors: Breast TNBC, Breast Luminal A, Breast Luminal B, Breast HER2 overexpressing, Melanoma, Pancreatic adenocarcinoma, NSCLC and Liver cancer. All compounds severely inhibit metastasis-cell invasion and -cell migration. At the same low drug concentration, they hardly affect cell proliferation (see “Supplementary Data”).

Another important observation that was made by the present inventors being that these 48 h incubation of anyone of the compounds at 5 µM concentration does not degrade the SKAI1BC lncRNA; Quantified by RT followed by Real-Time PCR with HMBS as endogenous control (data not shown). Without being bound by the theory, the inventors assume that compounds binding to the particular SKAI1BC lncRNA prevent the lncRNA annealing to the complementary sense DNA strand (at the KAI1 promoter region), and thus enabling the KAI1 gene to be transcribed. Alternatively, the compounds may interfere with the SKAI1BC lncRNA binding of an essential KAI1 gene/s-specific transcription factor, and in this way enhance KAI1 RNA transcription.

The metastasis-specific activities of these compounds, coupled to their lack of cytotoxicity, may also enable their use to prevent formation of disseminated tumor cells (DTCs). This latter is related to the recent discovery of biomarkers for generation of DTCs^22^. Obviously, such intervention is likely to be desirable in human tumors developing metastases early on after primary tumors’ formation. Ten additional solid cancers such as prostate, gastric, ovarian, colon, cervical, renal clear cell carcinoma, osteosarcoma, bladder, thyroid and LSCC cancers are known to be dependent on deficiency in KAI1/CD82 expression. Therefore, more tumors are likely to respond to these compound/s^6,7^. This also brings up the question whether SKAIBC lncRNA may serve as a biomarker for metastasis in a fraction of the solid human cancers. To the best of our knowledge, this is the first time that modeling of a large lncRNA (> 700 bp) secondary structure followed by its potential interaction with Inforna like compounds database, has led to identification of biologically active, potential small molecule drugs.

In view of the fact that until now, there are very few effective anti-metastasis drugs^23–27^, the potential importance of these compounds (which have been recently named Migrastatics) is clear.

## Methods

### Cell culture

The following cell lines were obtained from ATCC: TNBC MDA-MB-231, BT-549,

HCC70 and Hs578T; Melanoma SK-MEL-24, RPMI-7951 and MDA-MB-435;

Pancreatic cancer AsPC-1, BxPC-3 and CFPAC-1; NSCLC A-549, NCI-H1299, NCIH1975 and NCI-H2030; Liver hepatocarcinoma SK-HEP1 and C3A. They were routinely cultured according to ATCC “culture method” at 37 °C in 5% CO_2_ each in its recommended medium supplemented with 5–10% fetal bovine serum (FBS) (Biological Industries), 10 units/ml of penicillin and 50 µg/ml streptomycin.

### Modeling of the SKAI1BC lncRNA secondary structure and its potential interaction with Inforna compounds

The SKAI1BC as-lncRNA (792 nt) was folded using ScanFold 2.0^21,28,29^. The transcript was folded in 120 nt windows, 1 nt increments (step size = 1), and 37 °C temperature. The Gibbs free energies of the structures (ΔG_37_°) were compared to the average free energy of 100 randomized sequences. Structures containing base pairs with Z_avg_ values below − 2 were refolded with RNAfold in the ViennaRNA package^30^ using ScanFold-predicted base pairs as constraints to extend helices and remove isolated base pairs. The probabilities of secondary structural features were calculated using ProbScan in the RNA structure package^31^. Figures of RNA folding results were generated for publication using IGV-ScanFold^32^ and VARNA^33^. Inforna was queried with these structures to find compounds that bind to structural motifs with high fitness scores^15^. The following compounds were received from NCI and dissolved in DMSO to a final concentration of 0.05%.

Compound #1: NSC no. 50460.

Compound #2: NSC no. 50469.

Compound #3: NSC no. 57161.

Compound #4: NSC no. 63676.

Compound #5: NSC no. 364277.

### RNA extraction from cell culture

Total RNA of the different cultures was extracted using Quick-RNA™ MiniPrep Kit (Zymo, ZR-R1055) according to the manufacturer instructions. RNA concentration and RNA quality were determined by SpectraMax quick drop micro-volume spectrophotometer (Molecular Devices).

### Reverse transcription (RT)

Reverse transcription was carried out using RevertAid™ Premium kit (Thermo Fisher), using hexamer primers and according to the manufacturer instructions. Usually, 1 µg of RNA was reverse-transcribed in a 20 µl reaction volume. In order to identify promoter-spanning lncRNAs of antisense orientation (so it is not mixed with plausible upstream-initiating protein coding transcripts), total cellular RNA/nuclear RNA from the human tumor cell lines, were subject to primer-specific RT-PCR.

### Quantitative real-time PCR

RNA level was assessed using the TaqMan Gene Expression Assay (Applied Biosystems) and a QuantStudio 1 RT-qPCR System (Thermo Fisher) following the manufacturer-recommended procedures. TaqMan® PCR took place in 96-well reaction plates holding a volume of 10 µL in each well. The mixture consisted of TaqMan® Gene Expression Assay 20 × (Thermo Fisher Scientific), TaqMan® Universal PCR Master Mix 2 × (Thermo Fisher Scientific), and 30 ng of desired cDNA. The 2ΔΔ^CT^ method was used for quantification of the relative RNA expression applying HMBS as the normalizing housekeeping gene. Each sample was validated three times (n = 3).

### Compounds effect on KAI1 mRNA and SKAI1BC lncRNA level

Cells were treated with 5 µM of compounds #1–5 for 48 h and total RNA was extracted and reverse-transcribed to cDNA. Relative KAI1 mRNA expression, as well as SKAI1BC lncRNA, were quantified by Real-Time PCR with HMBS RNA as endogenous normalizing control.

### Cell migration assay

To study cell migration^34^, cells were treated with 5 µM of each of the compounds #1–#5) for 24 h. Then, ThinCert™ (Greiner Bio-One) cell culture inserts were placed in a multi well cell culture plate. Each insert contains a polyethylene terephthalate (PET) membrane at the bottom with a pore size of 8 µm that separates the upper from the lower compartment. Of the pre-treated cells, 100,000 serum-starved cells were seeded at the top of the insert in 200 µl serum free media, with or without 5 uM of the different particular compound. The lower compartment contains 10% FBS media as a chemo-attractant that may induce active migration of the seeded cells through the PET membrane. Adherent cells that migrate through the pores remain attached to the underside of the PET-membrane. After 24 h, the medium in the inserts and lower compartment was removed. To estimate the percentage of migrated cells, a Resazurin Cell Viability Assay was performed and compared to the mock control cells.

### Cell invasion assay

Cells were treated with 5 µM of each of the compounds (#1–#5) for 24 h. Cells were then seeded in CytoSelect (CBA-110, Cell Biolabs) transwell chambers for additional 24 h, while under starvation conditions, and in the presence of the compounds. Cells invasion from the upper chamber to the chemoattractant (10% serum) in the lower chamber were quantified using colorimetric assay and according to the manufacturer instructions^35^.

### Comparison of gene expression pathways affected in TNBC cells by compound #3 versus compound #5

TNBC MDA-MB-231 cells were grown in six well plates and treated with either 0.05% DMSO as a mock, or 5 µM of either Compound #3 or Compound #5. Forty-eight hours later, RNA was extracted from a six well plate using 1 ml of Trireagant (T9424) according to manufacturer’s instructions. RNA concentration and purity were measured with Nano drop to verify QC 1.8–2.2 for 260/230 and 260/280. RNA integrity was examined on Tapestation 2200 (Agilent) using RNA Tape and Buffer (Agilent RNA SCREEN TAPE 5067–5576, RNA SCREEN TAPE SAMPLE BUFFER 5067–5577) and confirmed to be > 9.5 for all samples.

Poly (A) selection was performed with kit NEX Poly (A) beads 2.0 (48 rxn)-NOVA512992 (Perkin Elmer) using 1.2 µg according to manufacturer’s instructions and eluted in 14 ul. Enriched RNA was used for RNA library preparation NEXTFLEX Rapid XP DNA-seq Kit (96rxn)-514,903 (Perkin Elmer) according to manufacturer’s instructions using 15 min fragmentation and 10 cycle amplification. cDNA was quantified using DSDNA HIGH SENSITIVITY 1000 RXN-DSDNA-H2 and quality was assessed with Tapestation (D1000 SCREEN TAPE 5067–5582 and D1000 REAGENTS 5067–5583) to determine band specificity and size.

Libraries were pooled and sequenced on a Hiseq (Illumina). Samples were demultiplexed using Bcl2FastQ and paired score quality was measured using fastqc and multiqc to be > 30 for > 87% of all samples.

Samples were aligned to Hg38 genome using STAR with default parameters. Mapping quality was examined using SeqMonk and > 85% were found to map to exons, with no rRNA or mtDNA contamination. Samples were quantified using htseqcount and compared using Deseq2 with default parameters. Pathways analysis was performed using ClusterProfiler tool, using genes with a ≥ 1.5-fold change and significance of 0.1FDR to obtain enriched GO terms and KEGG pathways^36–38^.

### Statistical analysis

Statistical analysis of mock-treated and compound-treated cells was carried out using a one-tailed t-test. The null hypothesis in each case was that the compounds had no effect on cell migration, invasion, or proliferation or KAI1 mRNA levels.

## Data availablity

All data generated or analyzed during this study are included in this published article, and its “Supplementary information files”. Some of the data can be found in our International Patent Application WO 2022/249192 A1.

### Supplementary Information

Supplementary Information.
