# Supplementary material for: Broad-spectrum metastasis suppressing compounds and therapeutic uses thereof in human tumors
Source: Sci Rep. 2023 Nov 21;13:20420. doi: 10.1038/s41598-023-47478-x (PMC10663508; doi:10.1038/s41598-023-47478-x)

**SUPPLEMENTARY DATA**

**Testing the compounds effect on proliferation of various cells lines**

In order to find out whether the effect of the compounds on metastases formation is genuine and not a reflection of cell growth inhibition, we tested the compounds’ effect on cell proliferation. For this purpose, cells were seeded evenly at the magnitude of about 10^5^ cells/ml into a 6-well plate. The cells were incubated with the medium alone or with the addition of 5uM of the different compounds. After 48 h of incubation the cells were harvested, trypan blue was added and the cells counted automatically using EVE cell counter (NanoEnTek). Each experiment was performed three times, and the average values calculated and used for the appropriate figures.

The effect of the disclosed compounds on proliferation of the, TNBC cell lines MDA-

MB-231, BT-549, MCF-7 (Luminal A),

ZR-75-30 (Luminal B) and SkBr3 (HER2+), the melanoma cell lines SK-MEL-24 and RPMI-7951, the NSCLC cell lines NCI-H1299 and NCI-H2030, the pancreatic cancer cell lines AsPC-1 and CFPAC-1, the liver cancer cell lines SK-HEP1 and C3A were tested as outlined above. Results show that compound #3 inhibited TNBC cell line MDA-MB-231 by 3%. Compound #4 boosted the same cell line growth by 10% **(Supplementary Data figure 1)**. Compound #1 inhibited melanoma cell lines SK-MEL-24 and RPMI-7951 by 3% and 8%, respectively. While compound #4 increased both cell lines growth by 11% **(Supplementary Data figure 3)**. Compound #5 inhibited pancreatic carcinoma cell lines AsPC-1 and CFPAC-1 by 5% and 7%, respectively **(Supplementary Data figure 5)**. Compounds #1-#3 inhibited the NSCLC NCI-H1299 cell line by 9%, 8% and 7%, respectively **(Supplementary Data figure 4)**. Similar results were also demonstrated in liver cancer cell lines SK-HEP1 and C3A (**Supplementary Data figure 6)**. So over all at the same low compounds concentration, which inhibit metastasis cell invasion and migration, they hardly affect cell proliferation.

**FIGURES LEGENDS**

**Supplementary Data Fig. 1: *Compounds effect on TNBC cell lines Proliferation***

MDA-MB-231, and BT-549 cells were counted and seeded evenly into 6-well plates. The cells were incubated with the medium alone or with the addition of 5uM of the different compounds. After 48 h of incubation, the cells were harvested and counted again to assess cell proliferation. Data are presented as the mean +/- standard error of the mean of three independent experiments.

**Supplementary Data Fig. 2: *Compounds effect on breast cancer cell lines proliferation***

MCF-7, ZR-75-30 and SkBr3 cells were counted, seeded evenly into 6-well plates, and then treated and processed as outlined above.

**Supplementary Data Fig. 3: *Compounds effect on Melanoma cell lines proliferation***

SK-MEL-24 and RPMI-7951 cells were counted and seeded evenly into 6-well plates, and then treated and processed as outlined above.

**Suplementary Data Fig. 4: *Compounds effect on NSCLC cell lines proliferation.***

NCI-H1299 and NCI-H2030 cells were counted and seeded evenly into 6-well plates, and then treated and processed as outlined above.

**Supplementary Data Fig. 5: *Compounds effect on pancreatic cancer cell lines proliferation***

AsPC-1 and CFPAC-1 cells were counted and seeded evenly into 6-well plates, and then treated and processed as outlined above.

**Supplementary Data Fig. 6: *Compounds effect on liver cancer cell lines proliferation***

SK-HEP1, and C3A cells were counted and seeded evenly into a

6-well plates, and then treated and processed as outlined above.


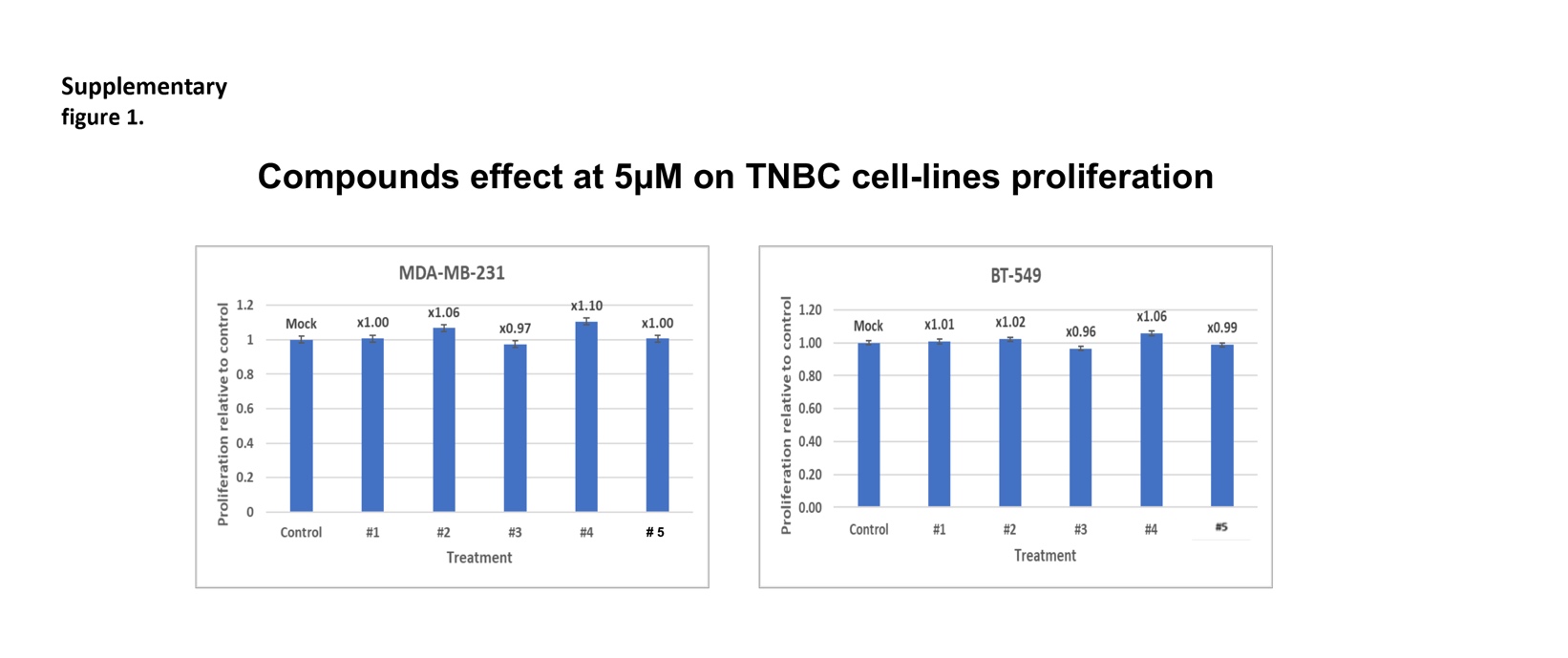


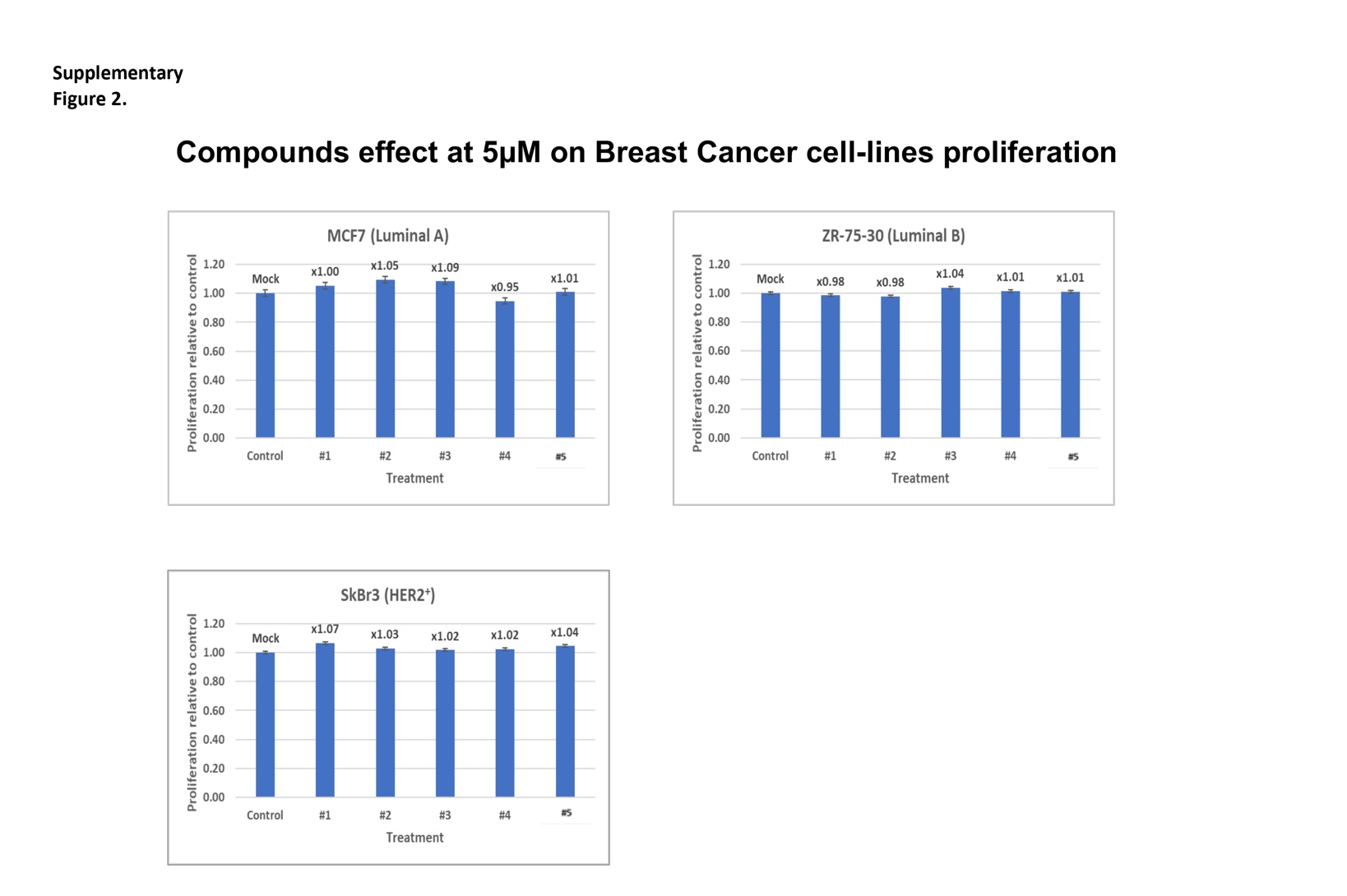


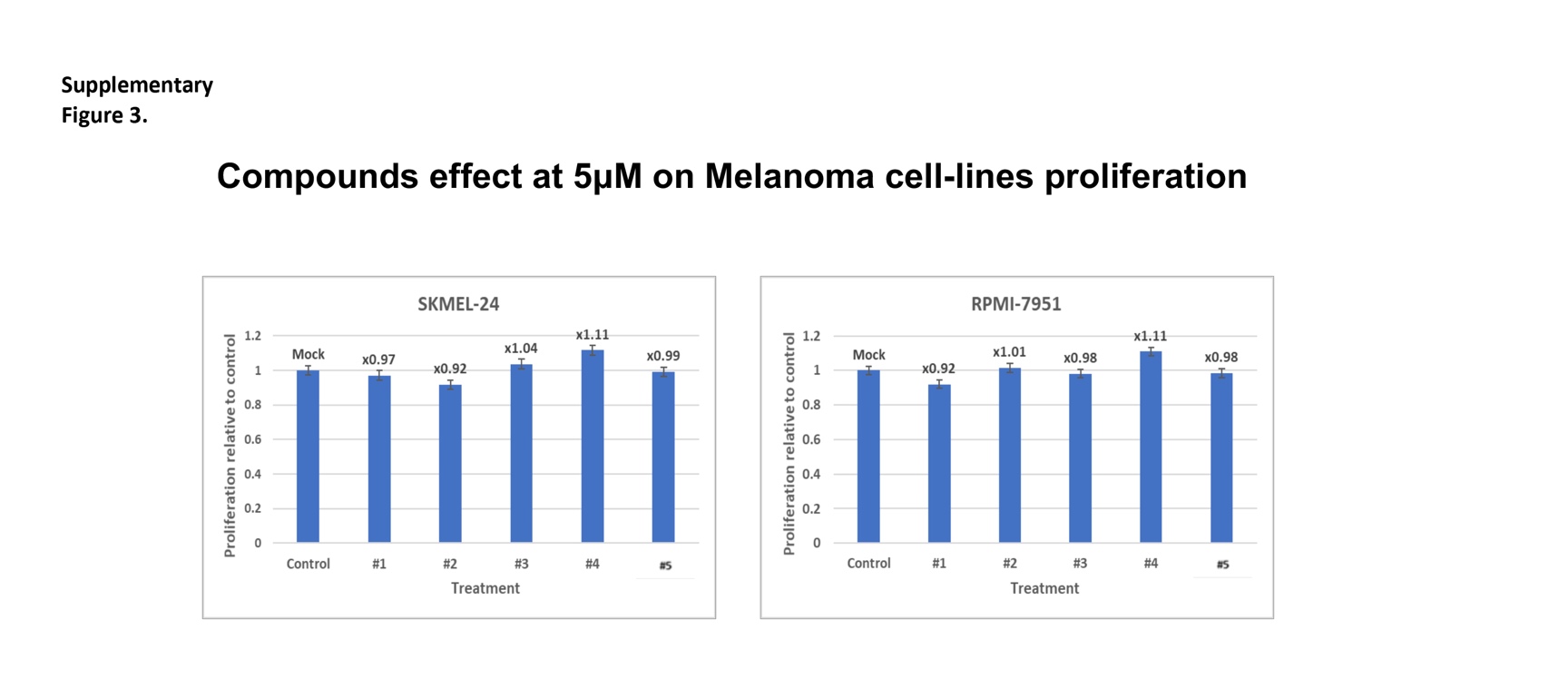


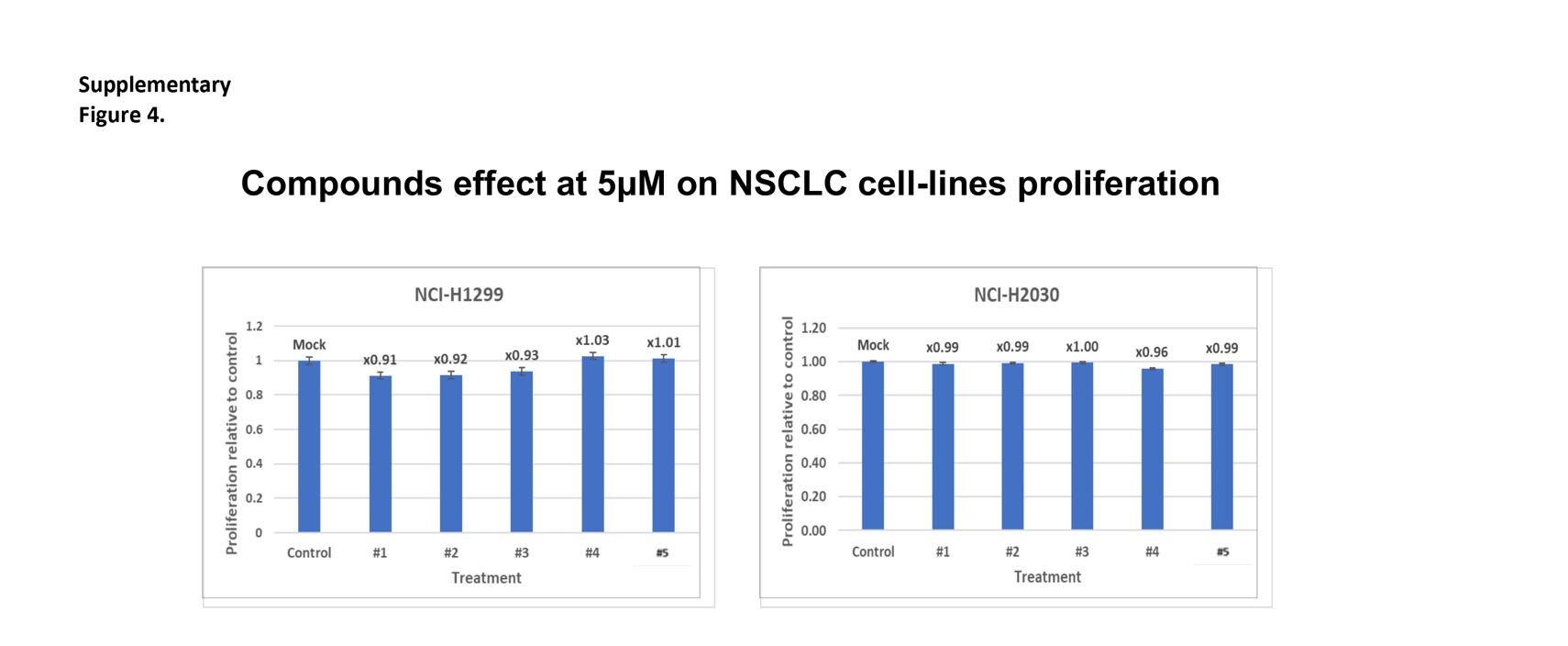


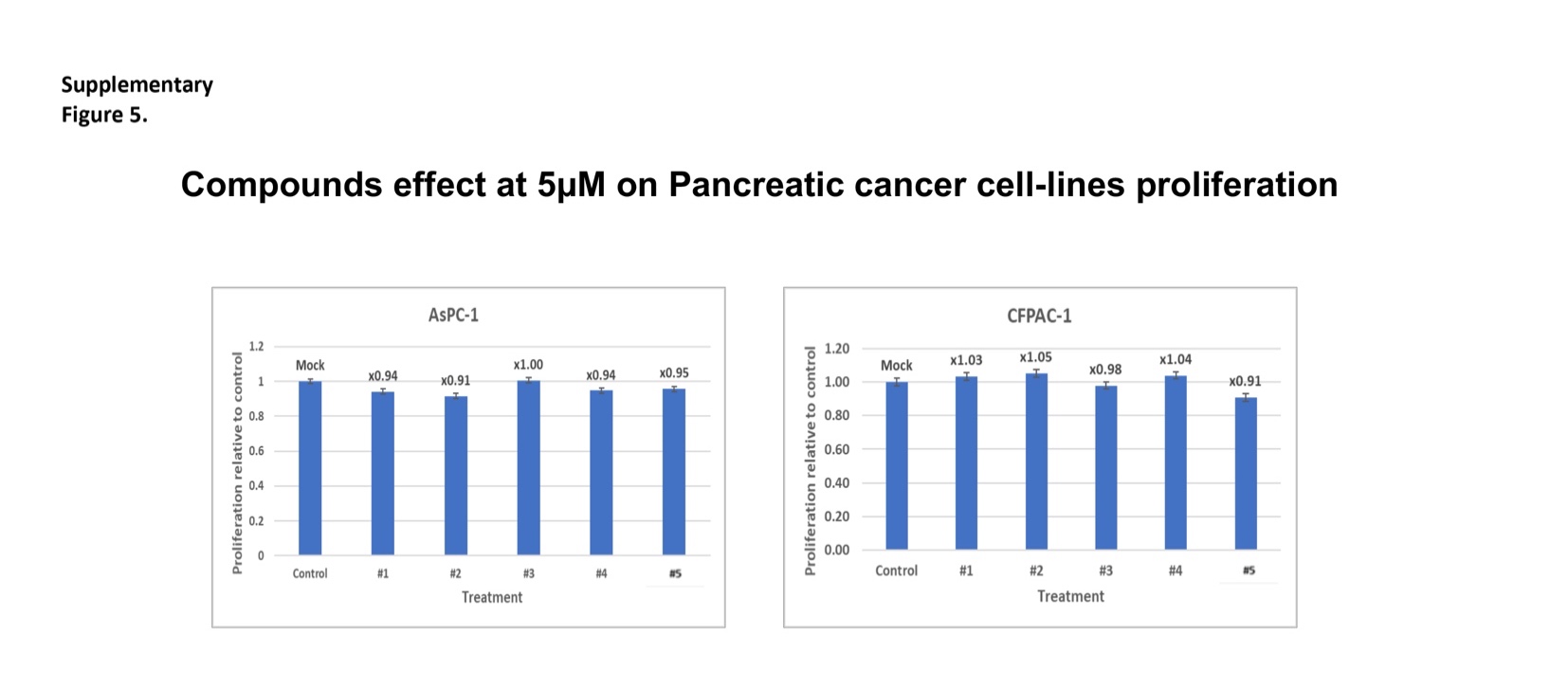


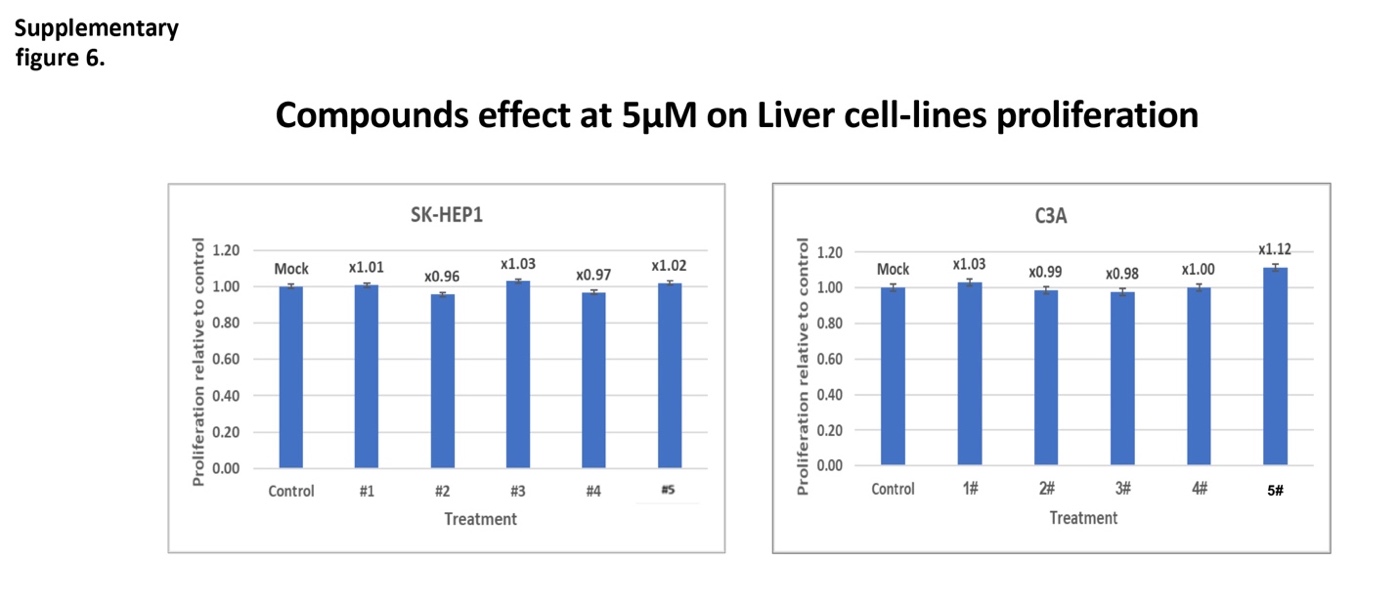

Supplement: Supplementary file 1 — Supplementary Information. [file 41598_2023_47478_MOESM1_ESM.docx]
